# *Coxiella burnetii* in free-living feral pigs (*Sus scrofa*) in Brazil

**DOI:** 10.1590/0074-02760250284

**Published:** 2026-04-20

**Authors:** Jorlan Fernandes, Wania Guimarães dos Santos, Matheus Ribeiro da Silva Assis, Martha Lima Brandão, Marcione Brito de Oliveira, Jairo Dias Barreira, Dominique Elvira Souza Freitas, José Luís Passos Cordeiro, Luiz Flamarion Barbosa de Oliveira, Elba Regina Sampaio de Lemos

**Affiliations:** 1 Fundação Oswaldo Cruz-Fiocruz, Instituto Oswaldo Cruz, Laboratório de Hantaviroses e Rickettsioses, Rio de Janeiro, RJ, Brasil; 2 Fundação Oswaldo Cruz-Fiocruz, Vice-Presidência Produção e Inovação em Saúde, Rio de Janeiro, RJ, Brasil; 3 Fundação Oswaldo Cruz-Fiocruz, Instituto Oswaldo Cruz, Laboratório de Biologia e Parasitologia de Mamíferos Silvestres Reservatórios, Rio de Janeiro, RJ, Brasil; 4 Universidade Federal do Rio de Janeiro, Museu Nacional, Departamento de Vertebrados, Mastozoologia, Rio de Janeiro, RJ, Brasil; 5 Universidade Federal do Estado do Rio de Janeiro, Departamento de Microbiologia e Parasitologia, Rio de Janeiro, RJ, Brasil; 6 Fundação Oswaldo Cruz-Fiocruz, Fortaleza, CE, Brasil

**Keywords:** Coxiella burnetii, feral pigs, wild boars, ticks, endosymbiont, Q fever, coxiellosis

## Abstract

**BACKGROUND:**

*Coxiella burnetii*, the etiological agent of coxiellosis in animals and Q fever in humans, is a zoonotic pathogen of global relevance that can infect a wide range of species. Although several domestic and wild animals are involved in the natural cycle, the role of wildlife hosts remains poorly understood.

**OBJECTIVES:**

Our study aimed to investigate the presence of *C. burnetii* in feral pigs hunted in Brazilian Pantanal wetland.

**METHODS:**

In this study, 36 free-living feral pigs legally hunted in Mato Grosso State, Brazil, were sampled. Sera were tested by enzyme-linked immunosorbent assay (ELISA), and spleen, liver, sera and tick samples were analysed by polymerase chain reaction (PCR).

**FINDINGS:**

Serological evidence of exposure was detected in 22.2% [8/36; 95% confidence interval (CI): 11.7% - 38.1%], while *C. burnetii* DNA was found in one spleen sample (1/36 - 2.8%; 95% CI: 0.1% - 14.5%). Only *Coxiella*-like endosymbiont was detected in *Amblyomma sculptum* ticks (9/23 - 39.13%; 95% CI: 22.2% - 59.2%).

**MAIN CONCLUSIONS:**

These results represent the first detection of *C. burnetii* in free-living feral pigs in Brazil and suggest potential exposure of this invasive mammal species to the pathogen. The findings underscore the need for broader surveillance of *C. burnetii* at the wildlife-livestock-human interface in Brazil.

*Coxiella burnetii* is a Gram-negative bacterium that causes disease in humans (Q fever) and animals (coxiellosis). Q fever is usually asymptomatic or presents with mild flu-like symptoms, although infection may be severe, especially in individuals with underlying conditions such as immunocompromised and cardiac valve defects, which can develop serious complications like pneumonia, endocarditis and hepatitis.^1^ ^,^ ^2^ ^,^ ^3^ Coxiellosis causes similar clinical outcomes and pathologies in wild and domestic animals, especially in pregnant female, that may experience reproductive problems such as placentitis, abortion and low birth weight.^4^ ^,^ ^5^

Various mammals, birds and reptiles have been reported to be infected with *C. burnetii*, including both domestic species and wildlife.^6^ ^,^ ^7^ The primary route of infection are through inhalation of aerosols or dust contaminated with placental materials, faeces, or vaginal secretions from infected animals.^1^ ^,^ ^5^ ^,^ ^4^ Ticks are thought to play a pivotal role transmitting the pathogen in livestock and wildlife mammals by feeding on an infected host and subsequently passing it to the next host during later feedings.^6^ ^,^ ^7^ ^,^ ^8^ ^,^ ^9^ Also, feeding behavioural patterns could modulate exposure to *C. burnetii* in humans, through the consumption of raw meat and milk, and in wild animals indicating that predator-prey relationships, presumably by ingestion of these prey species.^1^ ^,^ ^6^ ^,^ ^7^ ^,^ ^10^ ^,^ ^11^

Wildlife species serve as force multipliers, thereby spreading the pathogen from infected environments to diverse and remote habitats. Furthermore, there is an increasing overlap between habitats of wild animals and human residential areas due to deforestation, land development, and food scarcity.^12^ ^,^ ^13^ ^,^ ^14^ ^,^ ^15^ This indicates that wild animals have the potential to contribute to the spread of *C. burnetii* among humans, domestic and production animals.^6^ ^,^ ^7^ ^,^ ^10^ ^,^ ^11^ Among wild animals, feral pigs and wild boars (*Sus scrofa*) are known to serve as reservoirs for *C. burnetii* in various countries.^16^ ^-^ ^23^

Feral pigs are distributed worldwide and have an extensive living range and movement patterns across the world.^16^ ^,^ ^24^ In Brazil, first records of feral pigs were reported in the 1960s and increased in the late 1980’s and early 1990’s, showing an accelerated increase in the last 30 years, and found in all six Brazilian biomes.^25^ ^,^ ^26^ ^,^ ^27^ Feral pigs, also known as “porco monteiro”, were introduced into the Pantanal biome (wetlands) as domestic pigs, that later become feral during the Paraguayan War (1865-1870), when farms were abandoned, and the animals escaped into the wild.^28^ Currently, it is estimated that there are approximately ten thousand feral pig herds widely distributed throughout the Pantanal’s landscapes where they apparently contribute to the conservation status of wildlife in the region and are of great importance to Pantanal culture, as there is a clear preference for hunting of feral pigs.^25^ ^,^ ^26^ ^,^ ^27^ ^,^ ^28^ ^,^ ^29^ Therefore, our study aimed to investigate the presence of *C. burnetii* in feral pigs legally hunted in Mato Grosso State, Brazil.

## MATERIALS AND METHODS

A cross-sectional study was conducted between November 2014 and October 2015, in the municipality of Barão de Melgaço, Mato Grosso State in the Northeastern Brazilian Pantanal, on a traditional cattle ranch (16º53’38.25”S, 55º54’24.98”W; Figure).^30^ Samples were obtained from freshly hunted animals, provided by local residents and ranch workers engaged in traditional subsistence hunting commonly practiced in the region.^29^ No animals were hunted or handled alive for research purposes, and all collections complied with ethical and legal regulations.

The project team did not handle live animals consequently ethical approval was not deemed necessary by Fiocruz Animal Use Ethics Committee (P.11/2025.1), SISBIO license 49647-1.

From each freshly hunted individual, blood, tissue fragments (liver and spleen), and ticks were collected after external inspection of the animals’ bodies. Blood (2-10 mL) were sampled in tubes without anticoagulants and subsequently centrifuged, tissues and ticks were stored individually and kept frozen at -20ºC until laboratory processing.

**Figure:**
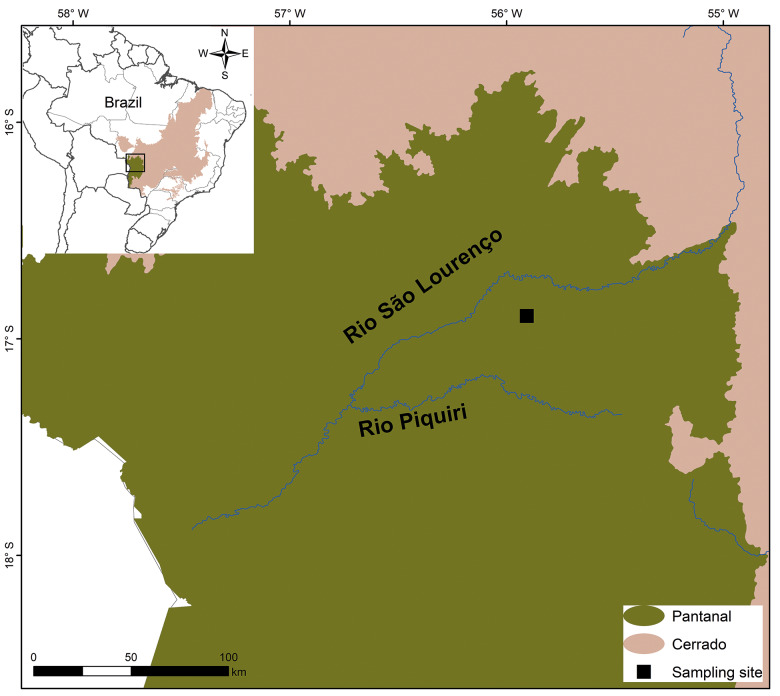
Map showing the data collection site of the feral pigs (*Sus scrofa*) on a traditional cattle ranch in the municipality of Barão de Melgaço, Mato Grosso, in the northeastern Pantanal, Brazil. Source: IBGE Biomes (Available from: https://www.ibge.gov.br/geociencias/cartas-e-mapas/informacoes-ambientais/15842-biomas.html).

Sera samples were tested using an indirect enzyme-linked immunosorbent assay (ELISA) for the detection of phases I and II antibodies directed against *C. burnetii* for the presence of antibodies to *C. burnetii* in multiple species (ID Screen® Q fever Indirect Multi-species; IDvet, Montpellier, France), in accordance with the manufacturer’s recommendations. Optical densities (OD) of the tested samples and positive and negative controls were measured by an ELISA plate reader at 450 nm. The OD ratio of the sample and positive control (S/P) was calculated for each sample as follows: [(ODsample - ODnegative) / (ODpositive - ODnegative)] × 100. Ratios were stratified as three different rising categories: samples with S/P < 40% were considered negative, samples with S/P between 40% and 50% were considered doubtful, samples with S/P > 50% were considered positive, according to the manufacturer’s recommendations. Any serum sample that was initially classified as “doubtful” was retested and, if resulting doubtful again, it was then considered as negative.

DNA was extracted from blood, tissue samples and tick specimens collected from feral pigs using QIAamp DNA mini kit (Qiagen, Valencia, CA, USA), following the manufacturers’ instructions. Ticks were individually washed twice with distilled water to remove any adherent host material, and engorged ticks were dissected using separate sterile forceps and scalpel blades to separate head and legs that were used for DNA extraction. Subsequently, feral pigs’ blood and tissue’s DNA were submitted to a conventional polymerase chain reaction (PCR) targeting the endogenous mammalian glyceraldehyde-3-phosphate dehydrogenase (GAPDH) gene, to ascertain the presence of amplifiable DNA and then submitted to PCR assays.^31^ Bacterial DNA was detected using *Coxiella* spp. and *C. burnetii*-specific primers, designed to amplify the 16S rRNA and the repetitive element IS1111 associated with the transposase gene, respectively. Controls and conditions for Nested-PCR standardised previously.^32^ ^,^ ^33^

Amplified products were separated by electrophoresis in 1.5% agarose gels stained with Gel Red Nucleic Acid Gel StainTM 10000× in DMSO (Biotium, Hayward, CA, USA) under 100 V/150 mA electric current for 60 min. The products of the PCR assays were purified using the ExoSAP-IT PCR Product Cleanup Reagent enzyme (Applied Biosystems, Foster City, CA, USA) and sequenced using the BigDye™ Terminator v3.1 Cycle Sequencing Kit (Thermo Fisher Scientific™, Waltham, MA, USA) and the ABI PRISM 310 DNA Analyzer (Applied Biosystems™, Foster City, CA, USA).

Taxonomic identification of the collected ticks was performed using a stereomicroscope (Olympus Corporation, Tokyo, Japan) and following previously described taxonomic keys for adult ticks.^34^ ^,^ ^35^ Ticks were also identified by molecular analysis and for this purpose, after nucleic acid extraction DNA samples were tested by PCR assay targeting a portion of the tick mitochondrial 16S rRNA gene, as previously described.^36^

## RESULTS

A total of 36 individuals were analysed for *C. burnetii* infection: 36 blood samples, 26 spleen and liver fragments. Antibodies to *C. burnetii* were detected in 22.2% (8/36; 95% CI: 11.7% - 38.1%) feral pigs (five males and three females) [Table and Supplementary data (Table)]. *C. burnetii*’ IS1111 partial gene was amplified in one spleen sample 2.8% (1/36; 95% CI: 0.1-14.5) (GenBank PX501780) of a non-reactive male feral pig hunted in 2014, showing identity values higher than 99% with *C. burnetii* (GenBank MN868471).

**TABLE:** Distribution of characteristics, prevalence of *Coxiella burnetii* infection for free-living feral pigs (*Sus scrofa*), Mato Grosso State, Brazil

Variable	N (%)	Seropositivity (%)	PCR (%)
Sex			
Female	14 (38.9)	3 (21.4)	0 (0.0)
Male	22 (61.1)	5 (22.7)	1 (4.5)
Year			
2014	23 (63.9)	5 (21.74)	1 (4.3)
2015	13 (36.1)	3 (23.1)	0 (0.0)
Tick infestation			
Yes	4 (11.1)	1 (25.0)	1 (25.0)
No	32 (88.8)	7 (21.9)	0 (0.0)
Total	36 (100.0)	8 (22.22)	1 (2.8)

Ticks were collected, by convenience, from four out of 36 (11.11%) animals. All 23 ticks (nine males and 14 females) collected were identified as adults *Amblyomma sculptum* specimens (GenBank PX455748 - PX455750). In the PCR-based screening assays for Coxiellaceae agents based on the 16S rRNA gene, 39.13% (9/23; 95% CI: 22.2% - 59.2%; GenBank PX455199 - PX455206) of the ticks (five males and four females) were positive showing high identity with *Coxiella*-like endosymbiont (CLE) from *A. sculptum* collected in Brazil (GenBank CP033868) [Supplementary data (Table)].

## DISCUSSION

*Coxiella burnetii* is an example of a pathogen that needs the One Health perspective considering the environmental risk associated with domestic, production and wild mammals, particularly in regions of nature, where the human population is in close contact with agriculture and, consequently, a higher proximity with livestock and wildlife. This is the scenario of Brazilian wetlands (Pantanal biome). The Pantanal biome is a large floodplain in South America that covers 160,000 km^2^. In this area, extensive livestock production is the main economic activity, characterised by livestock-wildlife interface.^37^ ^,^ ^38^ ^,^ ^39^ Cattle and horses share the same habitats as the abundant wildlife including feral pigs, and white-lipped peccary (*Tayassu pecari*), Southern coati (*Nasua nasua*), the ocelot (*Leopardus pardalis*) and the crab-eating fox (*Cerdocyon thous*).^39^ ^,^ ^40^ ^,^ ^41^ In this complex scenario *C. burnetii* represent a risk for humans and animals.

To date, only two studies have indicated the circulation of *C. burnetii* in Brazilian wetlands, in beef cattle (1.0% - 2/200) and in neotropical free-living brocket deer (*Mazama gouazoubira*) at lower rates (6.2% - 2/32) than the one described here (22.22%).^42^ ^,^ ^43^

The seropositivity found in our study is also higher than those reported previously for wild boars populations sampled in South Korea (14.6% - 142/975), Czech Republic (6% - 2/32), Spain (5.48% - 4/73), Portugal (1.1% - 4/358) and United States of America (0.47% - 5/1.063).^16^ ^,^ ^17^ ^,^ ^18^ ^,^ ^22^ ^,^ ^23^ No association was found between seroprevalence and sex, age or clinical signs, since animals showed no signs of disease. Coxiellosis causes similar clinical outcomes and pathologies in wild animals as it does in domestic animals and livestock, usually with no clear signs of infection, thus seroepidemiological studies that show the presence of infection are important in disease control, since wild ungulates can transmit the agent even while providing a seronegative result.^18^ ^,^ ^44^ In fact, here *C. burnetii* DNA was amplified from a spleen sample of a seronegative animal (2.80%) — indicating active infection before antibody production (seroconversion), or immune evasion leading to latency and localisation in the spleen — in a lower prevalence than described in wild boars from Spain (4.3% - 4/93) and slightly higher than the one reported in Italy (1.6% -1/63).^19^ ^,^ ^21^ The low PCR detection rate in means that the epidemiological role of feral pigs as an active reservoir should be interpreted with caution.

Ticks are known to play a significant role in the transmission of *C. burnetii* in wildlife populations, and they thrive in environments with abundant moisture and tall grass, which provide shade from the harsh sun.^7^ ^,^ ^8^ ^,^ ^9^ ^,^ ^16^ ^,^ ^45^ The presently observed high incidence of 39.13% of CLE, closely related but genetically distinct to *C. burnetii*, in *A. sculptum* ticks from feral pigs has also been demonstrated in a wide variety of Ixodidae ticks including *Amblyomma* spp.^6^ ^,^ ^7^ ^,^ ^46^ CLE are almost exclusively confined to ticks and pose a much lower infection risk to vertebrates, but has been commonly misidentified as *C. burnetii*. Based on the presence of IS1111 in both *C. burnetii* and the CLE, our group developed a nested-PCR assay, considering that IS1111 sequences in endosymbionts revealed to be genetically divergent, providing a specific *C. burnetii* detection, which resulted on a feral pig positive-PCR, but with no detection on *A. sculptum* ticks, including those collected from the PCR-positive pig.^6^ ^,^ ^33^

Although *A. sculptum* ticks have previously been found to be positive for *C. burnetii*, recent studies in feral pigs and associated ticks from São Paulo State, Brazil, no blood or tick samples showed to be positive in the qPCR for *C. burnetii* based on the IS1111 gene.^33^ ^,^ ^45^ Indicating that ticks may not be the main source of infection for feral pigs in Brazil. *C. burnetii* is probably far more frequently transmitted through the airborne route or consumption than through tick bites. Feral pigs are omnivores, meaning they have a diverse diet, opportunistically exploiting food resources according to availability during different seasons. Livestock farming is believed to buffer seasonal environmental fluctuations for feral pigs, providing artificial water sources, cattle carcasses for food. Thus, there are reports of predation of newborn calves and lambs, all of which could result in *C. burnetii* transmission.^28^ ^,^ ^30^ Additional studies involving feral pigs are needed to allow a better understanding of prevalence and transmission routes in Brazilian wetland ecosystems.

Considering that *C. burnetii* infections in wild mammals have been associated with infertility, abortion, stillbirth, endometritis, and mastitis, the detection of *C. burnetii* presented in this study should serve as a warning to professionals working in conservation programs for sensitive species, anticipating prey-to-predator spillover events, which have had significant implications.^5^ ^,^ ^11^ As an integral part of the trophic structure of Pantanal communities, feral pigs are important prey for predators in the food chain, such as the jaguar (*Panthera onca*), is important to evaluate the role of trophic webs as determining factor in *C. burnetii* enzootic cycle in a natural ecosystem.^28^ ^,^ ^47^

Feral pigs are a source of meat and fat, providing food security for the Pantanal community; hunting and castration activities strengthen social bonds of the local population. Handling infected animals can be an important source of *C. burnetii* infection for human and domestic animals like dogs. In Queensland almost one in five pig-hunting dogs (18.3%; 19/104) were seropositive to *C. burnetii*. One of the highest recorded in Australia, indicating that feral pigs and dogs could potentially be sources of *C. burnetii*.^48^ Although the zoonotic disease risk posed by feral pigs is poorly understood, our findings indicate that hunters should be aware of the risk of exposure to Q fever during hunts.

This study provides the first detection of *C. burnetii* in free-living feral pigs in Brazil, indicating that this invasive and exotic species may be exposed to the pathogen. While the limited sample size and the small number of PCR-positive animals require cautious interpretation, the results highlight the importance of further investigations with larger sample sizes to assess the epidemiological role of feral pigs in the transmission of *C. burnetii*. These findings support the need for continuous One Health surveillance at the wildlife-livestock-human interface.

## SUPPLEMENTARY MATERIALS

Supplementary material

## Data Availability

The sequences generated during the current study are available in GenBank under the following accession numbers: PX501780, PX455199 - PX455206 and PX455748 - PX455750.
