# Supplementary material for: Coxiella burnetii in free-living feral pigs (Sus scrofa) in Brazil
Source: Mem Inst Oswaldo Cruz. 2026 Apr 20;121:e250284. doi: 10.1590/0074-02760250284 (PMC13102456; doi:10.1590/0074-02760250284)
Supplement: Supplementary material [file 1678-8060-mioc-121-e250284-s1.pdf]

TABLE

Screening for *Coxiella burnetii* antibodies and DNA in 36 sera samples and 26 tissues of free-ranging feral pigs (*Sus scrofa*) and associated 23 ticks from Mato Grosso State, Brazil

	Sus scrofa					Tick (*Amblyomma sculptum*)	
			PCR assay		ELISA	PCR assay P/N (%)	
	Year	Gender	IS1111	16 S rRNA	(IDVet)	IS1111	16 S rRNA
LF5811	2014	M	NEG	NEG	NR	NEG	**2/4 (50.0)**
LF5812	2014	M	NEG	NEG	NR	-	-
LF5813	2014	F	NEG	NEG	NR	-	-
LF5814	2014	M	NEG	NEG	NR	-	-
LF5816	2014	M	NEG	NEG	NR	-	-
LF5818	2014	F	NEG	NEG	NR	-	-
LF5819	2014	M	NEG	NEG	NR	-	-
LF5820	2014	M	NEG	NEG	**R**	-	-
LF5821	2014	M	NEG	NEG	NR	-	-
LF5822	2014	M	NEG	NEG	NR	-	-
LF5823	2014	M	**POS**	NEG	NR	NEG	**3/9 (33.33)**
LF5824	2014	F	NEG	NEG	**R**	NEG	**0/2 (0.0)**
LF5825	2014	F	NEG	NEG	NR	NEG	**4/8 (50.0)**
LF5826	2014	F	NEG	NEG	NR	-	-
LF5827	2014	M	NEG	NEG	NR	-	-
LF5828	2014	M	NEG	NEG	NR	-	-
LF5829	2014	M	NEG	NEG	NR	-	-
LF5830	2014	M	NEG	NEG	NR	-	-
LF5831	2014	M	NEG	NEG	NR	-	-
LF5832	2014	M	NEG	NEG	NR	-	-
LF5833	2014	M	NEG	NEG	**R**	-	-
LF5835	2014	M	NEG	NEG	**R**	-	-
LF5836	2014	M	NEG	NEG	NR	-	-
LF5840	2015	M	NEG	NEG	**R**	-	-
LF5841	2015	M	NEG	NEG	**R**	-	-
LF5842	2015	F	NEG	NEG	**R**	-	-
LF5843*	2015	F	NEG	NEG	NR	-	-
LF5844*	2015	M	NEG	NEG	NR	-	-
LF5845*	2015	M	NEG	NEG	NR	-	-
LF5848*	2015	F	NEG	NEG	NR	-	-
LF5849*	2015	F	NEG	NEG	NR	-	-
LF5850*	2015	F	NEG	NEG	NR	-	-
LF5852*	2015	F	NEG	NEG	**R**	-	-
LF5853*	2015	F	NEG	NEG	NR	-	-
LF5854*	2015	F	NEG	NEG	NR	-	-
LF5855*	2015	F	NEG	NEG	NR	-	-
**PREVALENCE**			**2.80%**		**22.22%**		**39.13%**

*Only blood was collected; M: male; F: female; NR: nonreactive; R: reactive; NEG: negative; POS: positive.
